# Human Umbilical Mesenchymal Stem Cells-Seeded Bladder Acellular Matrix Grafts for Reconstruction of Bladder Defects in a Canine Model

**DOI:** 10.1371/journal.pone.0080959

**Published:** 2013-11-20

**Authors:** Haichao Yuan, Yue Zhuang, Ju Xiong, Wei Zhi, Liangren Liu, Qiang Wei, Ping Han

**Affiliations:** 1 Department of Urology, West China Hospital, Sichuan University, Chengdu, Sichuan, P.R. China; 2 Department of Rheumatology, West China Hospital, Sichuan University, Chengdu, Sichuan, P.R. China; 3 Department of Gynaecology, West China Second Hospital, Sichuan University, Chengdu, Sichuan, P.R. China; 4 Laboratory of Stem Cell and Tissue Engineering, State Key Laboratory of Biotherapy and Regenerative Medicine Research Center, West China Hospital, Sichuan University, Chengdu, Sichuan, P.R. China; Instituto Butantan, Brazil

## Abstract

**Background:**

The goal of this study was to explore the feasibility of utilizing human umbilical mesenchymal stem cells (HUMSCs)-seeded Bladder acellular matrix graft (BAMG) for bladder reconstruction in a canine model.

**Methodology/Principal Findings:**

HUMSCs were isolated from newborn umbilical cords and identified by flow cytometry. Partial cystectomy was performed in the experimental and control group. Bladder defects were repaired with HUMSCs-BAMG in the experimental group and repaired with unseeded-BAMG in control group. The implanted grafts were harvested after surgery. H&E and immunohistochemistry staining were performed to evaluate the regeneration of the bladder defect. Primary cultured HUMSCs displayed typical fibroblast morphology with spindle-shaped. Flow cytometry indicated that these cells were positive for CD105 (97.3%) and CD44 (99%), but negative for CD34 (2.8%), CD31 (2.1%), and CD45 (1.7%). Immunohistochemistry staining showed that a multilayered urothelium and well-developed smooth muscle were observed at 12 weeks in experiment group. In contrast, multilayered urothelial tissues were also observed at 12 weeks in group B, but well-developed smooth muscle bundles were observed.

**Conclusions/Significance:**

Our preliminary results demonstrate that UMSC-seeded BAMGs are superior to unseeded BAMGs to promote the regeneration of bladder defects. Our findings indicated that HUMSCs may be a potential cell source for bladder tissue engineering.

## Introduction

The repair of bladder defects caused by trauma or tumors is often problematic and poses a serious challenge for urological surgeons [1]. The development of tissue engineering techniques will bring new opportunities for bladder reconstruction [2]. These techniques involve seeding biomaterial scaffolds with appropriate cells in the laboratory and implanting them in vivo to repair or regenerate damaged tissue [3].

Certain studies have reported that transplantation of biomaterial seeded with autologous urothelial and smooth muscle cells could allow for the regeneration of a functional bladder in several animal models [4]–[6]. However, the use of autologous cells from patients with invasive bladder cancer or neurogenic bladders may eventually result in the reoccurrence of a diseased bladder state and a decline in urodynamic function during treatment [7].

Therefore, identifying a suitable cell source is a major challenge for cell therapy and tissue engineering. In addition to fulfilling the function of the reconstructed tissue, low immunogenicity is needed for clinical applications. Among the various types of cell sources, mesenchymal stem cells (MSCs) have drawn attention because they are characterized as undifferentiated cells, they are able to self-renew with a high proliferative capacity, and they possess a mesodermal differentiation potential [8], [9]. Currently, autologous adult MSCs, which can be easily harvested from various tissues such as bone marrow [10], adipose tissue [11] and muscle tissue [12], have been the main source of MSCs. However, the use of autologous adult MSCs is not always acceptable due to the high degree of viral exposure and the significant decrease in the cell number and the proliferative/differentiation capacity with increasing age [13], [14].Moreover, adult MSCs require painful invasive harvest; numbers are limited and their stem properties do not last for too long in vitro. Because of the disadvantages associated with autologous adult MSCs, it is essential to find an alternative source of MSCs.

In 2003, Mitchell et al. [15] reported the successful isolation of MSCs from porcine and human umbilical cord tissue by explant culture. Umbilical mesenchymal stem cells (UMSCs) can also be differentiated into adipocytes, osteoblasts and smooth muscle cells [16]–[18]. Umbilical cords can be collected at a low cost and provide an inexhaustible source of stem cells. Substantial numbers of UMSCs can be harvested within several passages without the need for long-term culture and extensive expansion ex vivo [19]. Moreover, the harvesting procedure of UMSCs is not invasive or painful, there is no donor site morbidity, and there is no ethical controversy related to the harvest of the resident stem cells. More interestingly, preliminary studies have shown that UMSCs do not express MHC II molecules, and the expression of MHC I molecules is also low [20]. Furthermore, MSCs, which may have immunosuppressive and immunomodulatory effects, evoke only minimal immune reactivity [21]–[23]. Clinically, the immunomodulatory properties of MSCs can be used to enhance engraftment and to reduce the incidence of graft versus host disease (GvHD) after transplantation [24]. Therefore, UMSCs may become an ideal source of allogeneic cell transplantation.

The bladder acellular matrix grafts (BAMG) is collagen-based xenogenetic biomaterial [25]. After a series of physical and chemical processes, the cells and antigens of the bladder can be eliminated, while their framework can be partly or completely retained. Therefore, BAMGs have good biocompatibility and reasonable mechanical strength, but they are associated with little or no immune rejection. Previous investigations have demonstrated the feasibility of using BAMGs for bladder reconstruction [25], [26].

In the present study, we isolated and cultured HUMSCs in vitro and seeded them onto BAMGs to repair bladder defects in a canine model. Our study allowed for us to assess the feasibility of using HUMSCs-seeded BAMGs for bladder reconstruction and explore alternative cell sources for tissue engineering.

## Materials and Methods

### Ethics Statement

This study was performed with the approval of the institutional Animal Care and Use Committee of West China Hospital, Sichuan University. All of the experimental procedures were conducted according to local guidelines on the ethical use of animals and the National Institutes of Health “Guide for the Care and Use of Laboratory Animals” (NIH publication No. 80–23, revised 1996). Approval from the Institutional Review Board and the Ethics Committees of the West China Hospital was obtained for the collection of umbilical cords from human participants after obtaining written informed consent of participant's guardian.

### Experimental animals

35 adult beagle dogs weighing 10–15 kg were used in this study. Ten of these dogs were sacrificed to prepare the BAMGs. The remaining 25 dogs were divided into a sham group (5 animals) that did not undergo resection of the anterior aspect of the bladder, a control group (10 animals) that received unseeded BAMGs to repair a bladder defect and an experiment group (10 animals) that received UMSC-seeded BAMGs to repair a bladder defect.

### Bladder acellular matrix graft (BAMG) preparation

Fresh bladders were obtained from the sacrificed dogs, and the BAMGs were processed as described previously [27]. The mucosa of the bladders were manually removed and washed in distilled water in a stirring flask (200 rpm) for 2 days at 4°C, followed by treatment with 0.03% trypsin for 1 h. The bladder matrix was soaked for 3 days at 4°C in 0.5% Triton X-100 (Sigma) and 0.05% ammonium hydroxide. Lastly, the resultant matrix was disinfected using 0.1% PAA in 20% ethanol for 2 h, rinsed three times with sterile distilled water for 10 min and stored in 10% gentamycin sulfate at 4°C until use. The structural characteristics of the BAMGs were examined using hematoxylin and eosin (H&E) staining and scanning electron microscopy (SEM) to confirm the effectiveness of the preparation procedure.

### Isolation and culture of HUMSCs

Approval from the Institutional Review Board of the West China Hospital was obtained for the collection of umbilical cords from ten human subjects after obtaining informed patient consent. The umbilical cords were obtained after full-term births and placed in Hank’s Balanced Salt Solution (HBSS, Gibco) before harvesting the HUMSCs. After disinfection in 75% ethanol, the umbilical cord vessels were cleared off, the remaining mesenchymal tissue (in Wharton’s jelly) was diced into cubes of approximately 0.5 cm^3^ in Dulbecco’s Modified Eagle’s Medium (DMEM; low glucose) and then transferred into 25-cm^2^ flasks in a 37°C incubator at 5% CO^2^. These Wharton’s jelly tissues were completely immersed in DMEM-LG supplemented with 10% fetal bovine serum (FBS) to allow for the cells to migrate and attach to the plastic surface. The medium was changed every 3 days until the plastic-adherent cells reached near confluence. Then, the cells were passaged with trypsin-EDTA.

### Flow cytometric analysis of HUMSCs

Third-passage HUMSCs were trypsinized and spun by centrifugation for 3 min at 1200 rpm. The pellet was resuspended in 50 µl phosphate-buffered saline (PBS) and incubated at 4°C for 30 min with the following cell-specific antibodies conjugated with fluorescein isothiocyanate (FITC) or phycoerythrin (PE): CD105, CD44, CD34, CD45 and CD31 (BD Biosciences Franklin Lakes, NJ, USA) in the dark. The labeled cells were washed twice with 1 ml PBS, resuspended with PBS, diluted in 200 µl PBS and then analyzed with a FACScan flow cytometer (Beckman Coulter Epics XL, Miami, FL).

### Seeding the HUMSCs onto the BAMGs

Before cell seeding, the BAMG sheet was cut into fragments of approximately 2×2 cm. For the control group, the BAMGs were incubated in DMEM-LG+10% FBS without HUMSCs prior to implantation. For the experiment group, the prepared HUMSCs were seeded on the BAMGs. Third-passage HUMSCs were seeded onto the outside of the BAMG at a concentration of 10×10^6^ cells per square centimeter in a flask filled with DMEM-LG+10% FBS at 37°C in 5% CO^2^ and 95% humidity for 10 days prior to implantation. The medium was changed daily for 24 h. The HUMSCs -seeded BAMGs were examined using SEM.

### Bladder reconstruction

Under anesthesia, a 7-cm lower midline incision was made in the abdomen of each dog. Once the dome of the bladder was exposed, the anterior aspect of the bladder (40% of the bladder) was resected with electrocautery. In the sham group, the anterior aspect of the bladder was not resected. In the control group, the unseeded BAMG was sutured as a patch onto the dome of the bladder using running 5–0 Vicryl sutures in 10 dogs. Prolene marking sutures were placed at the three, six, nine and 12 o’clock positions to rule out variation in the tissue harvest. The graft was covered with omentum to improve tissue vascularization. In the experiment group, the HUMSCs-seeded BAMGs were sutured in the remaining 10 dogs using the same method and the cell-seeded side of scaffold faces to the bladder lumen during the surgery. The bladder was tested for leakage by instillation of saline solution. When anastomosis was satisfactory, the fascia, subcutaneous tissue and skin were closed with absorbable sutures. An 8 F catheter was used and maintained for 7 days for postoperative bladder drainage. All of the animals were treated with intramuscular Gentamycin (5 mg/kg) after surgery for 7 days.

### Histopathological examination and Immunohistochemistry

The animals in each group were divided over five time points and killed at 1, 2, 4, 8 and 12 weeks post-implantation. After euthanization, Bladder tissue specimens were isolated immediately following euthanasia and fixed in 10% neutral buffered formalin, dehydrated through an alcohol gradient, cleared, paraffin-embedded blocks and sectioned (8 µm). H&E staining was also performed according to a standard protocol to evaluate regeneration of the bladder. To identify urothelial and smooth muscle cell differentiation of the HUMSCs-seeded BAMG scaffolds in vivo; the tissue graft samples were analyzed by immunohistochemistry staining. The urothelial cell layers were identified using a cytokeratin (AE1/AE3) monoclonal antibody (Sigma, St. Louis, MO), and smooth muscle cells in the regenerating bladder were identified using anα-smooth muscle actin (α-SMA) monoclonal antibody (Sigma, St. Louis, MO).

## Results

### Morphological Features and Identification of cultured HUMSCs

**Figure 1:**
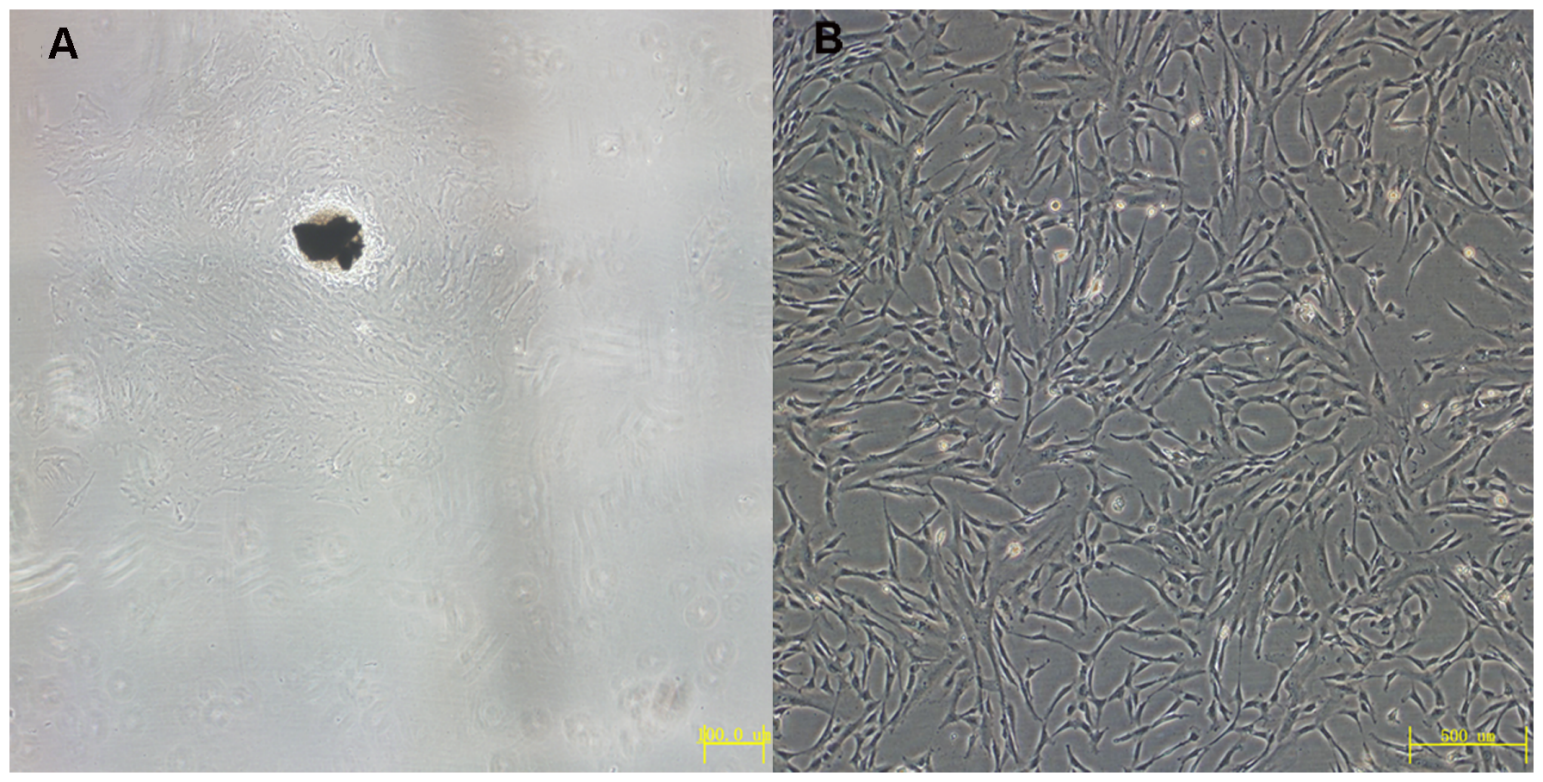
Morphology of HUMSCs. A the adherent cells the cells had migrated out of umbilical cord at 3 days in primate culture.(magnification×40) B the first passage cells displayed typical fibroblast morphology with a spindle-shape after incubation for 2 days. (magnification×100).

**Figure 2:**
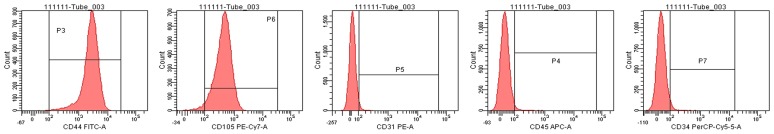
Flow cytometric analysis showing the immunophenotype of the second passage HUMSCs. The cells were positive for CD105 (97.3%) and CD44 (99%), but negative for CD34 (2.8%), CD31 (2.1%), and CD45 (1.7%).

After 3 days in culture, the cells had migrated out of the Wharton’s jelly tissues, and the nonadherent cells were rinsed from the culture flask (Fig 1A). When the adherent cells reached 80% confluence at 7 days and were passaged with trypsin-EDTA, the passaged cells displayed typical fibroblast morphology, with a spindle shape after incubation for 2 days, and they proliferated rapidly (Fig 1B). Flow cytometry showed that the adherent cells were positive for CD105 (97.3%) and CD44 (99%), but negative for CD34 (2.8%), CD31 (2.1%) and CD45 (1.7%), which was similar to the previous reports and indicated that these cells were mesenchymal stem cells from human umbilical cord tissue (Fig 2).

### The Character of BAMGs and Scaffold Seeding

**Figure 3:**
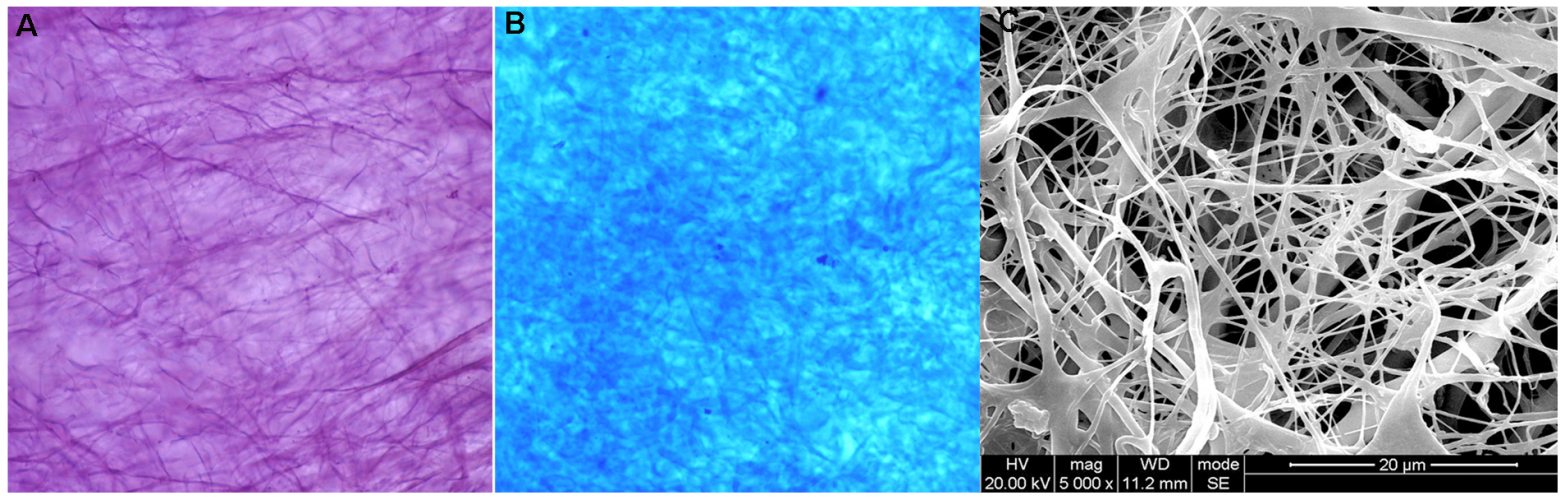
Micrographs of BAMG. A and B Photomicrograph of the BAMG stained with H&E and Masson trichrome stain (magnification×100). H&E and Masson trichrome staining showed no presence of cells after the decellularization process. C SEM micrograph of the BAMG showing reticular fibrous framework without residues of cells or cell fragments (magnification×5000).

**Figure 4:**
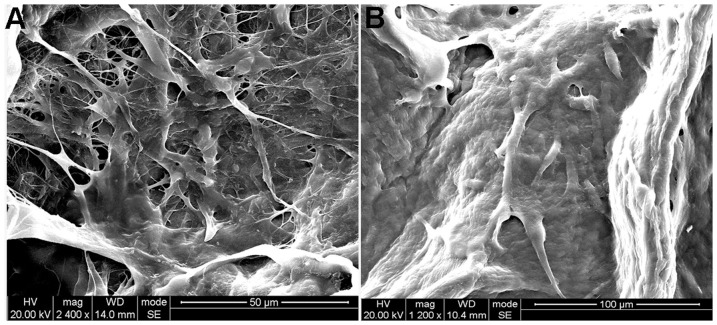
HUMSCs cultured on BAMG. A SEM showed HUMSCs migrated and proliferated actively in the three-dimensional fashion of BAMG (magnification×2400) at five days. B SEM showed HUMSCs overspread the surface of BAMG at 10 days (magnification×1200).

After processing, the BAMG appeared as a white, semitransparent film and the thickness is about 0.3–0.5 cm. The structure of the BAMG was an intact, reticular fibrous collagen framework without cells or cell fragments, as evidenced by H&E, Masson trichrome staining and SEM (Fig 3). After seeding, the BAMGs were maintained for 10 days to allow for cell adhesion. Prior to implantation, a 0.5-cm piece of each seeded BAMG was removed for testing to ensure enough seeding of the cells by SEM. SEM showed numerous HUMSCs migrated and proliferated actively in the three-dimensional fashion of BAMG at five days and overspread the surface of BAMG at 10 days (Fig 4).

### Surgical Outcomes and Histological Evaluation

All of the dogs recovered well after the surgery and survived until euthanized. No significant postoperative complications, such as infection and urinary leakage, were encountered in each group and all of the dogs voided spontaneously after the removal of their catheters.

Gross inspection showed that mild adhesions with the perivesical tissue were present in all of the animals. In three groups, the grafts could be distinguished from the host bladder before two weeks. After four weeks, the grafts were covered by soft, vascularized connective tissue and the graft borders could not be identified easily. There were no stones, tumors or diverticulum formations in any of the animals.

**Figure 5:**
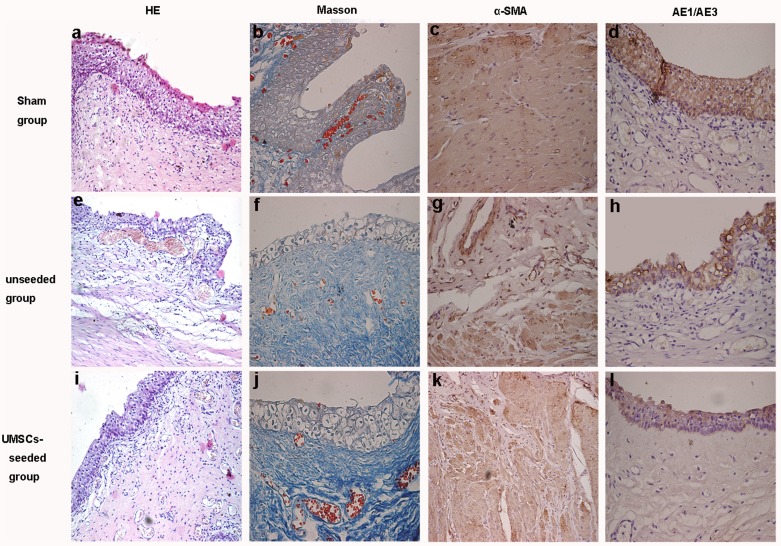
Histologic analysis of reconstructed bladder at 12 weeks after implantation. (A, E and I) Hematoxylin–eosin stain (magnification×200); (B, F and J) Masson trichrome stain (magnification×200); (C, G and K) immunohistochemistry staining showed the smooth muscle cells to stain positively with α-SMA antibody (magnification×200); (D, H and L) immunohistochemistry staining showed the urothelium cell to stain positively with cytokeratin (AE1/AE3) antibody (magnification×200).

All of the retrieved bladder tissues were studied by routine histological evaluation and immunohistochemistry. A moderate inflammatory response was observed with granulocyte and macrophage infiltration around the graft site at one week after implantation in the control group and the experiment group. In the experiment group, the regeneration of new urothelial tissue could be observed at the periphery of the graft at two weeks. At four weeks, the luminal surface of the graft was covered with urothelium and muscle cells. We also observed a decrease in the inflammatory response and an increase in the number of blood vessels. At eight weeks, a multilayered urothelium covered the entire graft with visible neovascularization and organized smooth muscle bundles. At 12 weeks, complete layers of transitional epithelium and well-developed smooth muscle were formed. However, urothelium regeneration was observed at 4 weeks in the control group. At 12 weeks, the smooth muscle cells on the graft were organized, but they still did not form well-developed smooth muscle bundles. Immunohistochemical staining showed that the urothelium cells were stained positively with a cytokeratin (AE1/AE3) antibody, and the smooth muscle cells were stained positively with a α-SMA antibody. (Fig 5)

## Discussion

Conventional bladder reconstruction, which generally uses autologous gastrointestinal tissue tissue, is associated with several complications, such as perforation, infection, metabolic disturbance, excessive mucus production and malignant transformation [28], [29]. Over the past years, different approaches have been developed in attempt to overcome the problems associated with traditional bladder reconstruction. The development of tissue engineering techniques will offer a potential alternative and bring new hope for bladder reconstruction [2], [30].

Numerous investigators have also studied the functional regeneration of bladder and urethra defects with cell-seeded biodegradable materials and indicate that a cell-seeded scaffold can contribute to successful repair of bladder and urethral defects. Sharma AK et al. [31] explored the regenerative capacity of bone marrow derived mesenchymal stem cells (BMSCs) seeded on small intestinal submucosa (SIS) in a baboon model and showed that the BMSCs exhibited a typical bladder architecture by Trichrome staining. Quantitative morphometry analyses revealed muscle-to-collagen ratios of approximately 32% and 52% in the unseeded versus seeded animals, respectively. Simple cytometry indicated that the bladder had a greater capacity to recover in animals treated with BMSC-seeded SIS than animals treated with unseeded SIS. Zhu WD et al. [26] reported that BAMGs seeded with adipose-derived stem cells could promote regeneration of smooth muscle, urothelium and nervous tissue in a rabbit model. The study showed that cell-seeded BAMGs were more suitable for bladder reconstruction than unseeded BAMGs. Orabi H et al. [32] reported that BAMGs combined with autologous cells resulted in the development of normal-appearing urethral tissue layers over time and can be used to repair long urethral defects, whereas scaffolds without cells lead to poor tissue development and strictures. These studies have shown the feasibility and effectiveness of using cell-seeded biodegradable materials to repair bladder defects in several animal models.

In the present study, HUMSCs were successfully isolated and cultivated. The isolated cells formed a morphologically homogeneous population of fibroblast-like cells. Flow cytometric analysis showed that the HUMSCs were positive for CD105 and CD44, but negative for CD34, CD31 and CD45, which is consistent with previous reports [33], [34] and suggests that these cells have a mesenchymal origin. We then tested the feasibility of using HUMSCs-seeded BAMGs to repair bladder defects in a canine model. A multilayered urothelium and well-developed smooth muscle were observed at 12 weeks in experiment group. In contrast, multilayered urothelial tissues were also observed at 12 weeks in control group, but well-developed smooth muscle bundles were observed. Therefore, our study showed that UMSC-seeded BAMGs provide better support for the formation of complete layers of urothelium as well as the regeneration of smooth muscle compared with unseeded BAMGs, indicating that HUMSCs can contribute to improving the regenerative capacity of tissue-engineered bladder.

Recently, a variety of synthetic and organic matrices used to reconstruct damaged bladders in animal models have been reported [31], [35]–[38]. In the present study, we selected BAMG as a biomaterial to reconstruct the bladder. BAMG is derived from bladder tissue, and as an acellular extracellular matrix, BAMG contains collagen, glycosamino-glycans, glycoproteins and many types of cytokines, such as FGF, TGF-β, BMP-4 and VEGF, that induce the attachment, proliferation and differentiation of cells and promote angiogenesis [27]. Our previous studies indicated that BAMG consists mainly of collagen fibers, and it has good biocompatibility without cytotoxicity [39]. We therefore selected BAMG as a biomaterial for bladder reconstruction.

However, the mechanism of tissue-engineered bladder regeneration based on MSCs is unknown, and the role of HUMSCs in bladder regeneration has not been elucidated. The observed regeneration may be attributed to migration of the native bladder cells, or the implanted HUMSCs may differentiate into smooth muscle cells and urothelium in bladder microenvironment.

Our study only assessed the short-term effects of the use of a biomaterial scaffold. Long-term studies are needed to evaluate the functional effects of the biomaterial scaffold for bladder reconstruction. Additionally, further research should be performed to explore whether HUMSCs are superior to the other MSCs for regeneration of the bladder.

## Conclusion

Our preliminary results demonstrate that HUMSCs-seeded BAMGs are superior to unseeded BAMGs to promote the regeneration of bladder defects. Our findings indicated that HUMSCs may be a potential cell source for bladder tissue engineering.

## References

[1] Duel BP, Hendren WH, Bauer SB, Mandell J, Colodny A, et al (1996) Reconstructive options in genitourinary rhabdomyoscarcoma. J Urol 156: 1798–1804. 8863619

[2] Atala A, Bauer SB, Soker S, Yoo JJ, Retik AB (2006) Tissue-engineered autologous bladders for patients needing cystoplasty. Lancet 367: 1241–1246. 16631879 10.1016/S0140-6736(06)68438-9

[3] Atala A, Vacanti JP, Peters CA, Mandell J, Retik AB, et al (1992) Formation of urothelial structures in vivo from dissociated cells attached to biodegradable polymer scaffolds in vitro. J Urol 148: 658–662. 1322466 10.1016/s0022-5347(17)36685-5

[4] Oberpenning F, Meng J, Yoo JJ, Atala A (1999) De novo reconstitution of a functional mammalian urinary bladder by tissue engineering. Nat Biotechnol 17: 149–155. 10052350 10.1038/6146

[5] Yoo JJ, Meng J, Oberpenning F, Atala A (1998) Bladder augmentation using allogenic bladder submucosa seeded with cells. Urology 51: 221–225. 9495701 10.1016/s0090-4295(97)00644-4

[6] Zhang Y, Kropp BP, Lin HK, Cowan R, Cheng EY (2004) Bladder regeneration with cell-seeded small intestinal submucosa. Tissue Eng 10: 181–187. 15009944 10.1089/107632704322791835

[7] Dozmorov MG, Kropp BP, Hurst RE, Cheng EY, Lin HK (2007) Differentially expressed gene networks in cultured smooth muscle cells from normal and neuropathic bladder. J Smooth Muscle Res 43: 55–72. 17598958 10.1540/jsmr.43.55

[8] Pittenger MF, Mackay AM, Beck SC, Jaiswal RK, Douglas R, et al. (1999) Multilineage potential of adult human mesenchymal stem cells. Science 284: , 143–147. 10.1126/science.284.5411.143 10102814

[9] Barry FP, Murphy JM (2004) Mesenchymal stem cells: clinical applications and biological characterization. Int J Biochem Cell Bio l36: , 568–584. 10.1016/j.biocel.2003.11.001 15010324

[10] Zhang Y, Lin HK, Frimberger D, Epstein RB, Kropp BP (2005) Growth of bone marrow stromal cells on small intestinal submu-cosa: an alternative cell source for tissue engineered bladder. BJU Int 96: 1120–1125. 16225540 10.1111/j.1464-410X.2005.05741.x

[11] Zuk PA, Zhu M, Ashjian P, De Ugarte DA, Huang JI, et al (2002) Human adipose tissue is a source of multipotent stem cells. Mol Biol Cell 13: 4279–4295. 12475952 10.1091/mbc.E02-02-0105 PMC138633

[12] Yokoyama T, Huard J, Pruchnic R, Yoshimura N, Qu Z, et al (2001) Muscle-derived cell transplantation and differentiation into lower urinary tract smooth muscle. Urology 57: 826–831. 11306423 10.1016/s0090-4295(00)01083-9

[13] Rubinstein P, Rosenfield RE, Adamson JW, Stevens CE (1993) Stored placental blood for unrelated bone marrow reconstitution, Blood. 81: 1679–1690. 8096404

[14] Fehrer C, Lepperdinger G (2005) Mesenchymal stem cell aging. Exp Gerontol 40: , 926–930. 10.1016/j.exger.2005.07.006 16125890

[15] Mitchell KE, Weiss ML, Mitchell BM, Martin P, Davis D, et al (2003) Matrix cells from Wharton’s jelly form neurons and glia. Stem Cells 21: 50–60. 12529551 10.1634/stemcells.21-1-50

[16] Karahuseyinoglu S, Kocaefe C, Balci D, Erdemli E, Can A (2008) Functional structure of adipocytes differentiated from human umbilical cord stroma-derived stem cells. Stem Cells 26: 682–691. 18192234 10.1634/stemcells.2007-0738

[17] Wang L, Singh M, Bonewald LF, Detamore MS (2009) Signaling strategies for osteogenic differentiation of human umbilical cord mesenchymal stromal cells for 3D bone tissue engineering. J Tissue Eng Regen Med 3: 398–404. 19434662 10.1002/term.176

[18] Baksh D, Yao R, Tuan RS (2007) Comparison of proliferative and multilineage differentiation potential of human mesenchymal stem cells derived from umbilical cord and bone marrow. Stem Cells 25: 1384–1392. 17332507 10.1634/stemcells.2006-0709

[19] Fan CG, Zhang QJ, Zhou JR (2011) Therapeutic potentials of mesenchymal stem cells derived from human umbilical cord. . Stem Cell Rev. 7: 195–207. 20676943 10.1007/s12015-010-9168-8

[20] Cho PS, Messina DJ, Hirsh EL, Chi N, Goldman SN, et al (2008) Immunogenicity of umbilical cord tissue derived cells. Blood 111: 430–438. 17909081 10.1182/blood-2007-03-078774

[21] Nauta AJ, Fibbe WE (2007) Immunomodulatory properties of mesenchymal stromal cells. Blood 110: 3499–3506. 17664353 10.1182/blood-2007-02-069716

[22] Weiss ML, Anderson C, Medicetty S, Seshareddy KB, Weiss RJ, et al (2008) Immuneproperties of human umbilical cord Wharton's jelly-derived cells. Stem Cells 26: 2865–2874. 18703664 10.1634/stemcells.2007-1028

[23] Yoo KH, Jang IK, Lee MW, Kim HE, Yang MS, et al (2009) Comparison of immunomodulatory properties of mesenchymal st em cells derived from adult human tissues. Cellular Immunology 259: 150–156. 19608159 10.1016/j.cellimm.2009.06.010

[24] Polchert D, Sobinsky J, Douglas G, Kidd M, Moadsiri A, et al (2008) IFN-c activation of mesenchymal stem cells for treatment and prevention of graft versus host disease, . Eur J Immunol 38: 1745–55. 18493986 10.1002/eji.200738129 PMC3021120

[25] Merguerian PA, Reddy PP, Barrieras DJ, Wilson GJ, Woodhouse KA, et al (2000) Acellular bladder matrix allografts in the regeneration of functional bladders: evaluation of large segment (>24cm(2)) substitution in a porcine model. BJU Int 85: 894–8. 10792173 10.1046/j.1464-410x.2000.00513.x

[26] Zhu WD, Xu YM, Feng C, Fu Q, Song LJ, et al (2010) Bladder reconstruction with adipose-derived stem cell-seeded bladder acellular matrix grafts improve morphology composition. World J Urol 28: 493–498. 20091038 10.1007/s00345-010-0508-8

[27] Chun SY, Lim GJ, Kwon TG, Kwak EK, Kim BW, et al (2007) Identification and characterization of bioactive factors in bladder submucosa matrix. Biomaterials 28: 4251–4256. 17617449 10.1016/j.biomaterials.2007.05.020

[28] Golomb J, Klutke CG, Raz S (1989) Complications of bladder substitution and continent urinary diversion. Urology 34: 329–338. 2688258 10.1016/0090-4295(89)90435-4

[29] Kaefer M, Hendren WH, Bauer SB, Goldenblatt P, Peters CA, et al (1998) Reservoir calculi a comparison of reservoirs constructed from stomach and other enteric segments. J Urol 160: 2187–2190. 9817364

[30] Chung SY (2006) Bladder tissue-engineering: a new practical solution? Lancet 367: 1215–1216. 16631860 10.1016/S0140-6736(06)68481-X

[31] Sharma AK, Bury MI, Marks AJ, Fuller NJ, Meisner JW, et al (2011) A nonhuman primate model for urinary bladder regeneration using autologous sources of bone marrow-derived mesenchymal stem cells. Stem Cells 29: 241–250. 21732482 10.1002/stem.568

[32] Orabi H, AbouShwareb T, Zhang Y, Yoo JJ, Atala A (2013) Cell-seeded tubularized scaffolds for reconstruction of long urethral defects: a preclinical study. Eur Urol 63: 531–538. 22877501 10.1016/j.eururo.2012.07.041 PMC3554849

[33] Zhang H, Zhang B, Tao Y, Cheng M, Hu J, et al (2012) Isolation and characterization of mesenchymal stem cells from whole human umbilical cord applying a single enzyme approach. Cell Biochem Funct 30: 643–649. 22777760 10.1002/cbf.2843

[34] Batsali AK, Kastrinaki MC, Papadaki HA, Pontikoglou C (2013) Mesenchymal stem cells derived from Wharton's Jelly of the umbilical cord: biological properties and emerging clinical applications. Curr Stem Cell Res Ther 8: 144–155. 23279098 10.2174/1574888x11308020005

[35] Jack GS, Zhang R, Lee M, Xu Y, Wu BM, et al (2009) Urinary bladder smooth muscle engineered from adipose stem cells and a three dimensional synthetic composite. Biomaterials 30: 3259–3270. 19345408 10.1016/j.biomaterials.2009.02.035 PMC2744495

[36] Lai JY, Chang PY, Lin JN (2005) Bladder autoaugmentation using various biodegradable scaffolds seeded with autologous smooth muscle cells in a rabbit model. J Pediatr Surg 40: 1869–1873. 16338308 10.1016/j.jpedsurg.2005.08.028

[37] Liao W, Yang S, Song C, Li X, Li Y, et al (2013) Construction of ureteral grafts by seeding bone marrow mesenchymal stem cells and smooth muscle cells into bladder acellular matrix. . Transplant Proc. 45: 730–734. 23498814 10.1016/j.transproceed.2012.08.023

[38] Salem SA, Hwei NM, Saim AB, Ho CC, Sagap I, et al. Polylactic-co-glycolic acid mesh coated with fibrin or collagen and biological adhesive substance as a prefabricated, degradable, biocompatible, and functional scaffold for regeneration of the urinary bladder wall. J Biomed Mater Res A. 2013. Available from URL: http://www.ncbi.nlm.nih.gov/pubmed/23349110(DOI: 10.1002/jbm.a.34518) 10.1002/jbm.a.34518 23349110

[39] Han P, Song C, Wei Q, Yang YR (2007) Biocompatibility of Bladder Extracellular Matrix as Tissue Engineering Scaffold. Sichuan Da Xue Xue Bao Yi Xue Ban 38: 1009–1012. 18095609

