# Correction: Human Umbilical Mesenchymal Stem Cells-Seeded Bladder Acellular Matrix Grafts for Reconstruction of Bladder Defects in a Canine Model

**DOI:** 10.1371/annotation/20cbe947-8308-4bcc-8fcb-d77c4dc96a78

**Published:** 2013-12-03

**Authors:** Haichao Yuan, Yue Zhuang, Ju Xiong, Wei Zhi, Liangren Liu, Qiang Wei, Ping Han

The first and second authors, Haichao Yuan and Yue Zhuang, were not correctly indicated as having contributed equally to the article. 

